# Analysis of Graphomotor Tests with Machine Learning Algorithms for an Early and Universal Pre-Diagnosis of Dysgraphia

**DOI:** 10.3390/s21217026

**Published:** 2021-10-23

**Authors:** Louis Devillaine, Raphaël Lambert, Jérôme Boutet, Saifeddine Aloui, Vincent Brault, Caroline Jolly, Etienne Labyt

**Affiliations:** 1 CEA, University Grenoble Alpes, Leti, 38000 Grenoble, France; louis.devillaine@univ-grenoble-alpes.fr (L.D.); raphael.lambert@cea.fr (R.L.); jerome.boutet@cea.fr (J.B.); Saifeddine.ALOUI@cea.fr (S.A.); 2 CNRS, University Grenoble Alpes, University Savoie Mont Blanc, LPNC, 38000 Grenoble, France; caroline.jolly@univ-grenoble-alpes.fr; 3 CNRS, University Grenoble Alpes, Grenoble INP, LJK, 38000 Grenoble, France; vincent.brault@univ-grenoble-alpes.fr

**Keywords:** dysgraphia, pre-diagnosis, graphomotor, machine-learning, supervised, drawings, handwriting

## Abstract

Five to ten percent of school-aged children display dysgraphia, a neuro-motor disorder that causes difficulties in handwriting, which becomes a handicap in the daily life of these children. Yet, the diagnosis of dysgraphia remains tedious, subjective and dependent to the language besides stepping in late in the schooling. We propose a pre-diagnosis tool for dysgraphia using drawings called graphomotor tests. These tests are recorded using graphical tablets. We evaluate several machine-learning models and compare them to build this tool. A database comprising 305 children from the region of Grenoble, including 43 children with dysgraphia, has been established and diagnosed by specialists using the BHK test, which is the gold standard for the diagnosis of dysgraphia in France. We performed tests of classification by extracting, correcting and selecting features from the raw data collected with the tablets and achieved a maximum accuracy of 73% with cross-validation for three models. These promising results highlight the relevance of graphomotor tests to diagnose dysgraphia earlier and more broadly.

## 1. Introduction

At school, fine motor activities still represent between 18% and 47% of children’s activities [1] compared to 30 to 60% in 1992 [2], where 85% of this time is allocated to pure handwriting. As a major part of their learning process, handwriting is a very important ability that children need to master. It involves several different skills, as the children have to learn how to perform an accurate and complex motor task that has to be synchronized with their eye feedback [3]. Difficulties to perform handwriting in a convenient way are a concern because they may become a set back in the children’s schooling and lead to a degradation of self-esteem [4].

Despite correct learning and training, some children never master handwriting and their difficulties are actually pathologic. This disorder is called dysgraphia and affects between 5 and 10% of children [5,6,7,8]. In France, dysgraphia is diagnosed thanks to the Concise Evaluation Scale for Children’s Handwriting [6], also known as the BHK test, which is an adaptation from the Dutch BHK test [9]. The test consists of copying a text for five minutes, on a blank piece of paper. Only the first five lines are then rated by the examiner on the basis of thirteen quality criteria (size of the handwriting, gaps between letters, tremors, unreadable letters, etc.) and one speed criterion on the entire production (number of characters written in 5 min).

Although the BHK test is the reference test in France, when it comes to the diagnosis of dysgraphia, it suffers from several drawbacks. It contains a part of subjectivity as even trained examiners may give different scores, or even different diagnosis [6]: the inter-rater correlation in the French version of the BHK test ranges from 0.68 for beginner raters, to 0.90 for very experienced ones. Furthermore, the writing test requires two years of writing practice, which consequently delays the diagnosis to an age older than seven. Because handwriting difficulties do not resolve without remediation [4], the sooner we detect them, the lesser they will affect the lives of children [10]. Another disadvantage of the BHK lies in its language-dependency: because it requires an adaptation to each language, it cannot be used on every child, and thus leads to a multiplication of tests and a difficult transposition of results between them.

The aim of this study is to design a pre-diagnosis tool that would help the examiners and give a first insight into the writing difficulties children can have. Several attempts to create such a tool have already been published [11,12,13,14,15,16,17]. They generally involve graphic tablets that are able to record the path of the pen as a function of time, and usually a classification model that will do a prediction task, with the notable exception of [16]. They trained a neural network (RNN) to do a recognition task (recognition of a letter) and associated a failed recognition with poor handwriting. A successful classification has been performed by [11] but their database was limited to 27 typically-developing children (TD) and 27 children diagnosed with dysgraphia (DYS) using the HPSQ questionnaire [18]. [12] also obtained great results on a larger database of children (*n* = 298) with and without dysgraphia, by exploiting the kinematic parameters extracted from BHK texts, even though some methodologic issues have been raised by [19]. More recently, the same approach has been followed by [20], who differentiated qualitative dysgraphia and quantitative dysgraphia and analyzed a larger database of BHK tests (*n* = 580) of children from French schools and hospitals, getting more robust and more easily generalizable results. A summary of the scores of these models is presented in Table 1.

More recently, a study used the same kind of approach, but applied it to drawings instead of writing, for the detection of graphomotor difficulties [21]. They recorded drawings of graphomotor tasks and used a machine-learning model to determine the classification. Their focus was on features extraction, where they compared different categories of features (classical, modulation spectra, fractional order derivative and Q-factor wavelets transform) and their impact on the final classification. They used only one classifier and obtained 84% accuracy in the prediction of graphomotor difficulties.

**Figure 1:**
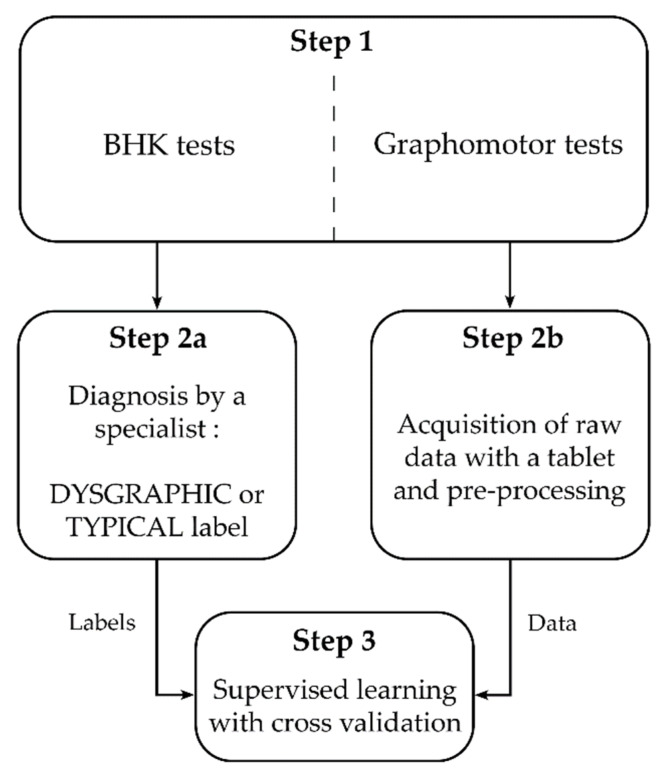
Main steps of the labelling, learning and validation procedure. Step 1 corresponds to the tests, performed at school or at the hospital. Then, the BHK (conducted on paper) are sent to the examiners to rate and create the labels in Step 2a, and the raw numerical data of the graphomotor tests are processed in Step 2b. Step 3 is the model evaluation.

This study intends to evaluate the possibility of using only graphomotor tasks, and no text-based test, for the detection of the developmental of dysgraphia -not only graphomotor difficulties- in order to avoid the drawbacks related to the dependence on the language and on the fact that the children are old enough to know how to write. Based on studies that showed that there are very informative data in the writing path of children performing simple shapes on a sheet of paper [11,22], we formulate the hypothesis that dysgraphia has an impact on the way children draw, even though writing and drawing are two distinct exercises [23]. The graphomotor tests consists in the reproduction by the subjects of several shapes on a graphic tablet: a total of six drawings or groups of drawings were analyzed, part of them stemming from the Developmental Test of Visual Perception version 2 [24]. Three hundred five children from 2nd to 5th grade performed the tests. Among the 305, all 43 children with dysgraphia, with 43 matching children without dysgraphia, were used as a database to train the machine learning model. The remaining 219 children are used as a reference database for computing age bias. Features reflecting both the kinematic and static characteristics of the children’s drawings were extracted from the graphomotor tests. The number of features is limited to those which are relevant to dysgraphia and may help in the building of an explanatory model of dysgraphia. After selecting the best combination of those features, we infer if the children display dysgraphia or not with a machine learning model, which is evaluated based on the cross-validation method (Figure 1). We evaluated nine models and three were selected for their best performances.

## 2. Materials and Methods

In this section, we present the characteristics of the participants before detailing our proposed procedure.

### 2.1. Participants

Three hundred five children from 2nd to 5th Grade (7 to 11 years old) took part in the study. This study was conducted in accordance with the Helsinki Declaration. It was approved by the Grenoble University Ethics Committee (CERGA, agreement 2016-01-05-79). The writing consent of all children’s parents and the oral consent of all children have been acquired. The database is presented in Table 2.

All children are French native-speakers. In order to obtain a database as exhaustive as possible, children have been recruited in 5 schools from the region of Grenoble (France), as well as in the Reference Center for Language and Learning Disorders of the Grenoble University Hospital (CRTLA, Centre Hospitalier Universitaire de Grenoble). Most of the children from schools were good writers but a few of them have been diagnosed with dysgraphia (4.9%). At the CRTLA, children were consulted because of handwriting difficulties that were becoming troublesome, and a majority (30 out of 40) of these children were indeed diagnosed with dysgraphia. Overall, there were 262 typically-developing children and 43 children with dysgraphia. Two of the children with dysgraphia have been diagnosed based on their speed only, and not because of their handwriting quality. Because of the variety of profiles leading to a diagnosis of dysgraphia, a Welch test was performed to compute the *p*-value of the distinguishability between the means of the different populations. As shown earlier, a gender effect was observed, with a prevalence of boys in the DYS group [6].

Children before 2nd Grade were not considered, as the BHK test cannot be used to rate people who have an experience of less than 2 years in handwriting [6] and are not present in Table 2. Twelve children who did not perform the tasks as they have been asked—lack of some shapes for instance—as well as children suffering from physical disabilities or neurological deficits have been excluded as well.

**Figure 2:**
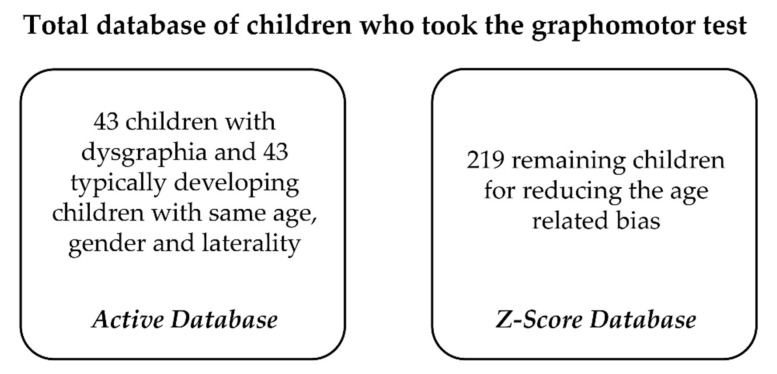
The database was divided into two sets. The *active database* is split into Train and Test for the supervised learning algorithm, and the *Z-score database* is only used as a reference, preventing any data leakage and overestimation of the performances of the model.

From this main database, we selected all 43 children with dysgraphia (13 from schools, 30 from the hospital), and 43 typical children paired to each of them. The pairing was determined by chronological age as follows: for each child with dysgraphia, among the typical children with the same age and gender, the one with the closest age was selected. Therefore, each pair has the same gender and laterality, and an average age gap of 15 days, the maximum being 76 days and the minimum 0 days. These 86 children constitute the *active database* used for the training and testing of the classification model. The remaining 219 typical children were used only to control any bias due to the children’s age. They constitute the *Z-score database* (Figure 2).

### 2.2. Approach & Procedure

All children took the test alone with an examiner, inside their school or the hospital, depending on where they were recruited. Two series of distinct tests were given to each child. The first ones are the graphomotor tests (described below), which are the tests that have been studied later on. Then, all the children took the BHK test following the procedure described in [6]. One or two expert examiners annotated the BHK tests in compliance with the Concise Evaluation Scale for Children’s Handwriting procedure. In this study, the only purpose of the BHK test was to have a real diagnosis and to correctly label children’s data for the supervised learning algorithms (see Figure 1). The recorded data of the BHK tests were not used in the machine learning algorithms.

**Figure 3:**
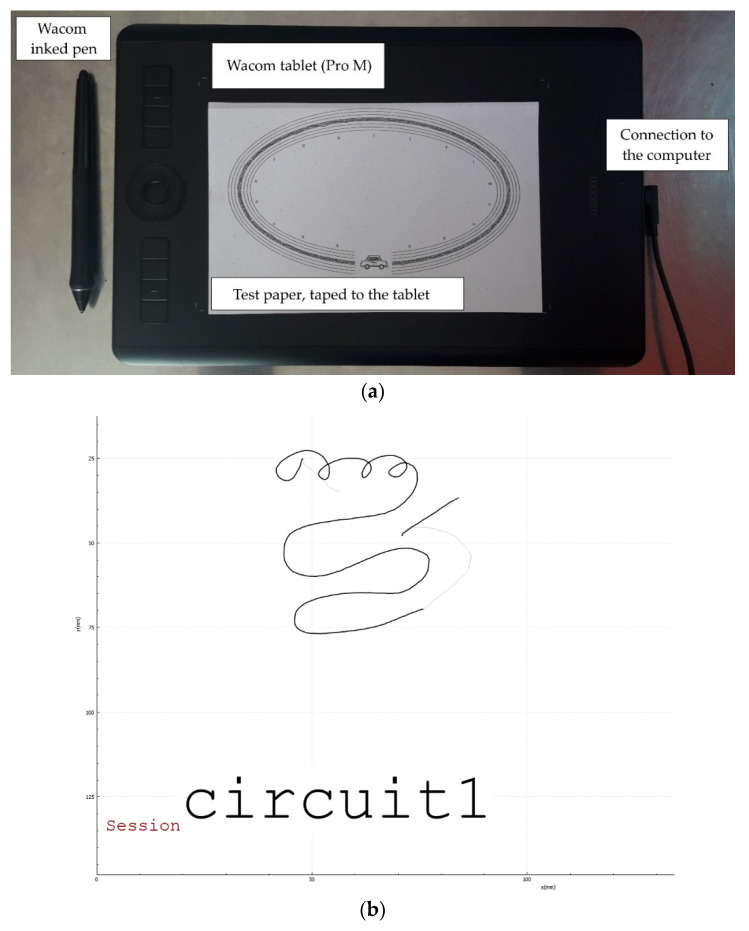
(**a**) Photo of the device used. They draw on a paper fixed on the tablet with an inked pen to reproduce normal writing conditions. The tablet here is the model used at school. The computer runs a software called GraphLogger, developed by the CEA, to record the drawings. (**b**) Example of the feedback of GraphLogger, seen by the examiner, for a scribble. The subjects do not see this screen. In black are the drawn parts, and in grey the projection on the tablet of the position of the tip of the pen when in air. Stimuli name is written (here it is *Circuit 1*).

The graphomotor tests, as well as the BHK test, were performed on a graphic tablet that records the x, y and z positions of the pen tip as a function of time, as well as pressure data (Figure 3a). Data were collected with a software called GraphLogger, developed by the CEA [20] (Figure 3b). Different models of graphic tablets were used at school (WACOM Pro M) and at the hospital (WACOM Pro L). A step of calibration has thus been necessary to be able to compare effectively the records coming from the two tablets: to check the written length calibration, different distances on the real written tracks made by the children on paper were measured and then compared to measures recorded by the computer to check the reliability of the tablets and software solutions. The spatial resolution of both tablets is 0.25 mm, and a new dot is considered every 5 min ms (200 Hz sampling rate).

Studies suggest that people write differently on paper than on screen [25], which led us to put a sheet of paper onto the tablet and an ink pen to better suit the usual writing conditions for the children. The children could write directly on the paper and had a visual feedback of their handwriting. All the data were processed with Python, using the following modules: scikit-learn [26], pandas [27] and mlxtend [28].

### 2.3. Tasks: The Graphomotor Tests

The graphomotor tests consist of 6 drawings, also called “stimuli”, which the children have to reproduce without a time limit. The first 6 drawings were extracted from the Developmental Test of Visual Perception [24], a test widely used in clinical context to evaluate “visual perception and visual-motor integration”. The last one, *The Loops*, can be used to evaluate dysgraphia levels as it is close to real handwriting [29].

We differentiated two kinds of graphomotor tests: the highlighting tests and copying tests. For the highlighting tests, the child had to trace directly on the drawing while trying to keep the pen in the thick grey path, without lifting the pen if possible. In the copying tests, the child must reproduce the drawings shown as accurately as possible. There are three of each, listed below.

Highlighting Tasks

**Figure 4:**
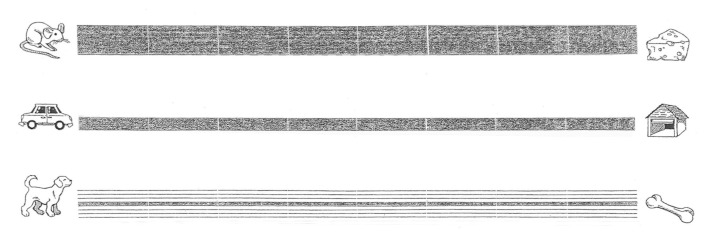
Circuit 1.

**Figure 5:**
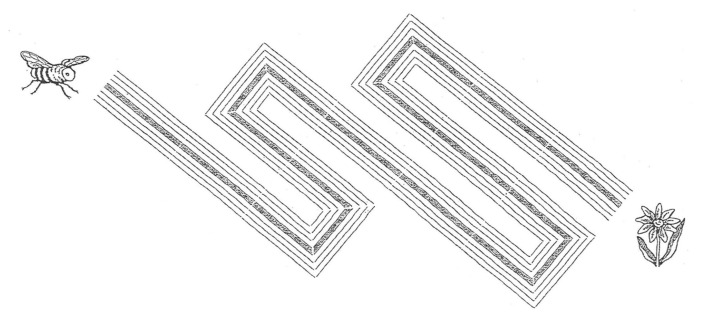
Circuit 2.

**Figure 6:**
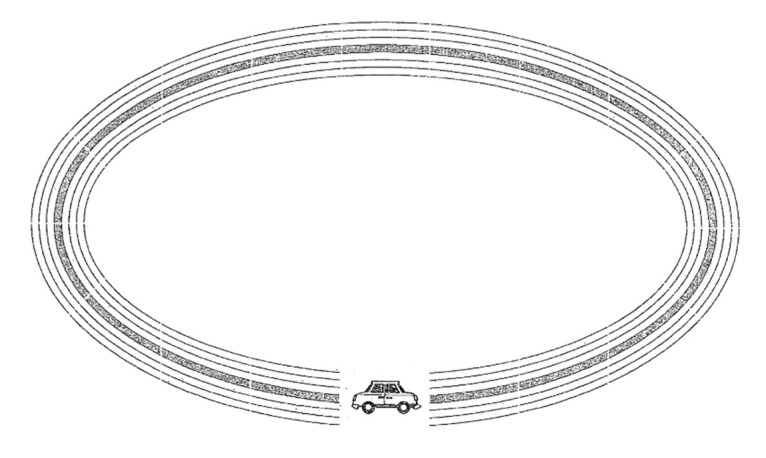
Circuit 3.

1. *Circuit 1* (known as the lines): the child has to link the left drawing to the right one. There are 3 lines, getting thinner and thinner, increasing the difficulty (see Figure 4).

2. *Circuit 2* (known as the labyrinth): likewise, the child links the bee and the flower. This task is more difficult to perform as there are a few abrupt turns (see Figure 5).

3. *Circuit 3* (known as the oval): the goal is the same, tracing a line from the left side of the car to the right side (see Figure 6).

Copying Tasks

**Figure 7:**
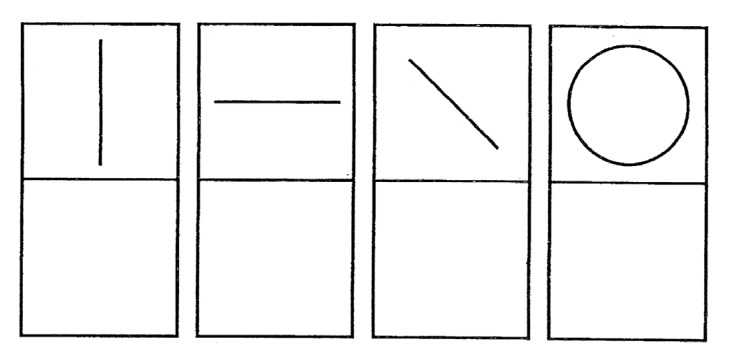
Shapes 1.

**Figure 8:**
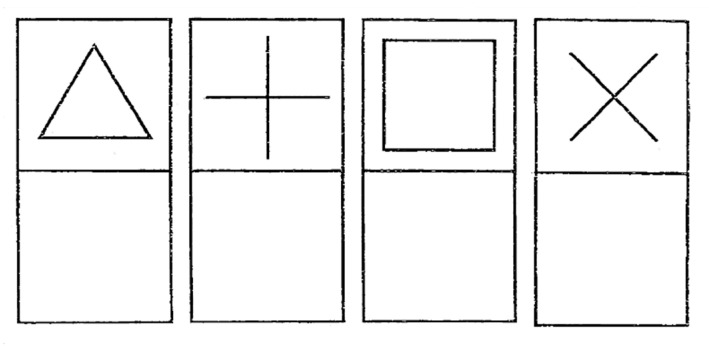
Shapes 2.

**Figure 9:**
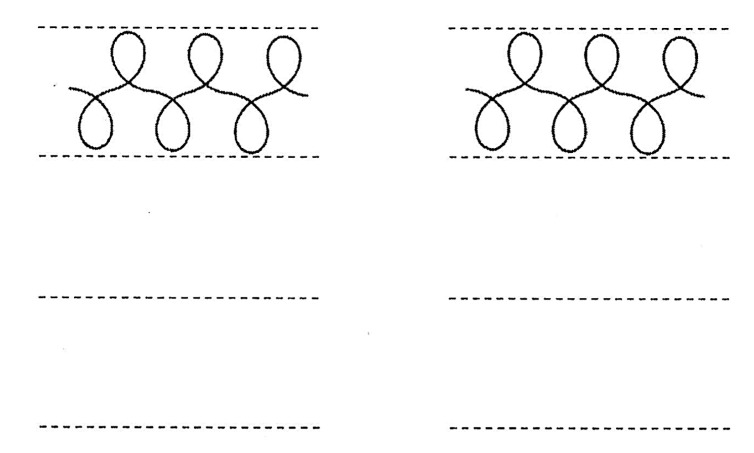
The Loops.

4. *Shapes 1*: there are four simple shapes (one vertical line, one horizontal line, one sloping line, and a circle) for the child to reproduce. They must be drawn in only one stroke (see Figure 7).

5. *Shapes 2*: four other shapes that are slightly more complex: a triangle, a plus sign, a square and a cross (see Figure 8).

6. *The Loops*: two series of six alternated loops. This is the hardest exercise of these graphomotor tests, as it requires great visual-motor coordination (see Figure 9).

### 2.4. Features Extraction

Many different characteristics can be extracted from the 6 stimuli, but only features that are relevant in regard to dysgraphia were used. They were computed from the data acquired during the tests, consisting of time series of the x (horizontal), y (vertical) and z (distance to the tablet up to 1 cm) coordinates of the pen tip, the pressure applied by the pen, and the different angles of the pen. Unfortunately, we were unable to calibrate pressure and angle data from the different tablets used, which led us to abandon the related features. However, the binary information stating whether the pen was on paper or in air (where no pressure is applied) was kept.

Before computing the features, a low-pass fourth order Butterworth filter was applied on the data to remove any noise that may be due to the tablets. We chose a cut-off frequency of 15 Hz, to keep most of the useful information about handwriting, which is comprised of around 5 Hz [30]. Two types of features are distinguished, general features and specific features. General features have been computed for each stimulus, which means that there are 6 occurrences of these features, 1 for each stimulus. Most of the general features used here are described in [20]. They are listed in Table 3.

Two features previously used in [37] for the evaluation of Parkinson’s Disease patients’ handwriting were added:

Rényi Entropy of order 2: delivers information about the entropy of the trajectory along x (resp. y or (x, y)). The entropy rate is linked to the uncertainty of the path, which manifests through a chaotic and unpredictable handwriting that is typical of children with dysgraphia. Their total Rényi Entropy should be higher in absolute value. The mean and standard deviation of the Rényi entropy of the standardized and normalized strokes was also extracted:

(1) $${\mathrm{RE}}_{2}=-\log\left(\sum_{i=2}^{N-1}p_{i}^{2}\right),$$

where N is the number of dots in the signal of interest (which is either x or y or the 2-dimensional time series of both x and y), and the *p* are the probabilities of each dot i to be where they are considering the handwriting is a strictly uniform movement.

Signal-to-Noise Ratio (SNR): reveals quick and unexpected movements from the children by comparing the trajectory along x (resp. y) to its smoothed version obtained by applying a basic low-pass-filter:

(2) $$\mathrm{SNR}=\frac{1}{N}\times \frac{1}{N\left(s\left[n\right]\right)},$$

where N is the number of dots in the signal s (which is either x or y).

Moreover, features specific to a particular stimulus were computed. An exhaustive list is given below:

Circuit 1

Mean squared error (MSE) of each line:

(3) $${\mathrm{MSE}}_{L}=\frac{1}{N}\sum_{i=1}^{N}{\left(y_{i}-y_{L}\right)}^{2},$$

where N is the number of dots for the line L, and y_L_ the height of the model line L.

Number of strokes per line:

The number of strokes, i.e., continuous writing between two pen lifts, for each line.

Duration of each line:

Time taken to draw each line.

Number of going backwards in a line:

The number of strokes beginning to the left (smaller x coordinate) of where the previous stroke ended, for each line.

Crossed distance before the first stop:

The percentage of line drawn before the first pen lift, for each line.

Standard deviation (Std) of the slopes:

The slope of each drawn line is computed via linear regression, and parallelism quantification is determined with the calculation of the Std of the 3 slopes.

Mean distance between the end of a stroke and the beginning of the next one.

The mean distance, across the 3 lines, of the gap between the end of a stroke and the beginning of the next one.

Circuit 2

Mean squared error on the long, short parts:

Same as *Circuit 1*, but the lines are the short and long parts of the circuit and are not horizontal.

Standard deviation of the slopes for the long, short parts:

Same as the Std of slopes for *Circuit 1*, but short parts and long parts are treated separately. Thus, there are 2 Stds computed: the Std of the slopes of the linear regressions of the 4 short parts, and the Std of the slopes of the linear regressions of the 5 long parts.

Percentage of drawing completed at first stop:

The percentage of completion of the circuit at first pen lift.

Number of lifts somewhere else than around a turn:

The number of pen lifts without a direction change between the previous and next stroke.

Mean and standard deviation of the quality of the angles:

For each angle, the Quality is computed.

(4) $$\mathrm{Quality}=1-\langle v_{b},v_{a}\rangle ,$$

where v_b_ is the direction of the line before the turn, and v_a_ is the direction after it. A Quality of 1 means the turn was 90°, which is perfect. The mean and Std Quality are computed for the whole stimuli.

Mean distance between the end of a stroke and the beginning of the next one:

Same as Circuit 1.

Circuit 3

Mean squared error to the perfect oval:

Same as *Circuit 1*, but with the oval as the reference.

Number of direction changes:

Number of times the general direction changes. For a normal drawing, it should be 4, as 4 main directions are considered. When there is tremor in the written path, it can increase.

Percentage of drawing completed at first stop:

Same as *Circuit 2*.

Rényi entropy adapted to the oval:

The Rényi entropy computed with Equation (1), with

(5) $$p_{i}=\frac{1}{\sqrt{200\pi }}e^{\frac{-{\left(d_{i}-r\right)}^{2}}{200}},$$

where d_i_ is the distance of the point i to the center, and r the radius.

Mean distance between the end of a stroke and the beginning of the next one:

Same as Circuit 1

Shapes 1

Duration of each shape:

Time taken to draw each shape.

Length of the path for each shape:

Total perimeter of each shape.

Index of curvature [22]:

Detailed in [20].

Horizontal, vertical diameter of the circle:

The length of the vertical and horizontal diameters of the drawn circle. For a perfect circle, the two should match.

Ratio between the horizontal and vertical diameter of the circle:

The ratio between the horizontal and vertical diameter of the circle. For a perfect circle, it should be equal to 1.

Shapes 2

Duration of each shape:

Time taken to draw each shape.

Length as the crow flies between the corners of the triangle and square:

The perimeter of the triangle formed using the three corners of the drawn triangle.

Length of the path for each shape:

Same as *Shapes 1*.

Ratio between the segments of the plus, of the cross:

Ratio of the length of the two lines composing the plus and the cross.

The Loops

Number of loops up, down, and total:

The number of loops combining the 2 parts of the drawing, separated in up loops, down loops and both. A normal drawing should have 6 up loops and 6 down loops (3 for each part).

Number of direction changes:

Same as *Circuit 3*. Will detect the wrong movements when, after doing an up loop, the subject is starting another before turning down to do the correct down loop.

Standard deviation of the width, of the height of the loops:

Each loop can have a different height and width, and the std of these values measure the inconsistency in forming the loops.

Ratio between the length of the trajectory dedicated to the loops and the total length (links between loops included):

The ratio between the loops themselves (the closed part of the loop), and the total trajectory, including the links between the loops.

Difference between the highest and the lowest point (links only, loops excluded):

The measure of general direction by calculating the difference between the highest point that is not in a closed loop, and the lowest.

Overall, it represents 345 features, general and specific features included.

### 2.5. Z-Score Computing

In order to apply the algorithms to the features, they need to be comparable. That is why they should evolve in the same range of values. To this end, features were standardized with a Z-score:

(6) $$\mathrm{Zscore}=\frac{\mathrm{Feature}-\mathrm{mean}\left(\mathrm{Feature}\right)}{\mathrm{standard}\;\mathrm{deviation}\left(\mathrm{Feature}\right)}.$$

However, children from the 2nd Grade and children from the 5th Grade do not have the same handwriting skills, given that the youngest ones are still beginners and their handwriting will improve throughout the years [35,38,39]. Their production thus cannot be compared directly and adding age as a feature does not entirely solve the problem.

Charles et al. took this concern into account when conceiving the BHK test [6]. The diagnosis indeed depends on the score of the child as well as its grade. Deschamps et al. thus proposed to implement a moving Z-score [20] that takes age into consideration during the standardization of the features. Instead of computing the Z-score over the entire database, the children were only compared to those who were utmost 6 months younger or older than them. The same method was used in this study.

The only issue with the Z-score as a data transformation is that it may lead to data leakage. If each data is transformed using the mean and standard deviation of all data from the same age range, when dividing into train and test sets, some information from the train set will be present in the test set, which can lead to the overestimation of classification results. To prevent this, only the data from the *Z-score database* were used as a reference for computing the Z-score. The data used for both the train and test set were the *active database* (Figure 2). Thus, no data from the train set were used for any transformations of the test set.

### 2.6. Preprocessing Steps

**Figure 10:**
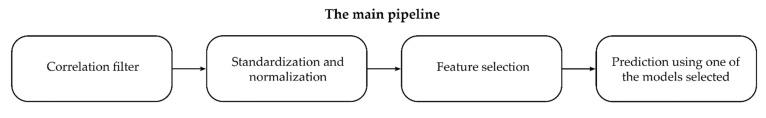
The main pipeline. The evaluation was performed through cross validation: firstly, the database was randomly split between train and test sets, then this whole pipeline was fit using the train set, and evaluated using the test data.

Considering that there is a total of 345 features, with only 86 data, a feature selection step must be conducted. The number of features should be reduced to the minimum, while keeping a sufficient amount of features to be able to efficiently perform the classification. To ensure the correct encapsulation of data, a pipeline containing 4 steps was created (Figure 10):

First, the removal of features that are too correlated. Above a certain threshold of correlation between 2 features, only 1 is kept. This threshold was set to 85%.

In the second step, the data were normalized and standardized.

In the third step, the feature selection was performed using an estimator which assigns importance to each feature. By selecting a threshold for this estimator, the number of features can be reduced. In our studies, we used either a linear SVM (C = 0.1) or an Extra Trees classifier with 100 estimators, and we selected 10 features.

Finally, the estimator for the prediction. Because we have a labeled database, we worked with supervised machine learning algorithms for our study. Several classes of algorithms have been used in the literature [11,12,13,14,15,16,40], therefore 9 models were tested, with different sets of hyperparameters, to select the best ones for each model.

The whole pipeline was then evaluated by a 5-fold cross validation. To avoid overestimation due to a particularly lucky cut for 1 cross validation, 100 iterations of cross validation with different random seeds for the splitting were performed. It is important that the preprocessing steps never used test data along with the train data, which is why we cannot undertake cross validation only on the final classifier that will determine the classification, but on the whole pipeline. Thus, for each fold, the train/test separation was conducted before any pre-processing step.

**Figure 11:**
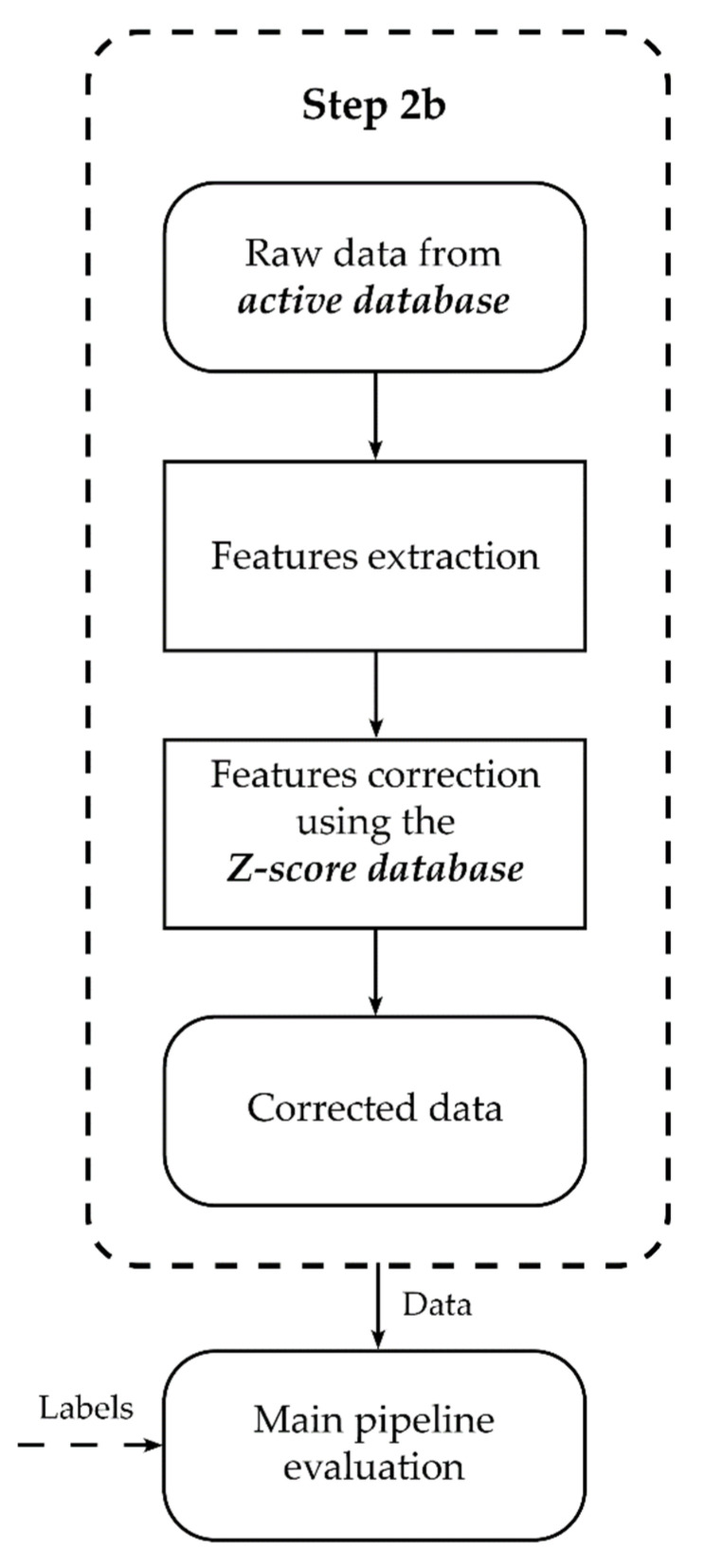
Summary of pre-processing. Starting from the raw data, features are extracted and corrected. The final data is then given to the main pipeline (Figure 10) and undergo more preprocessing (feature selection).

Figure 11 is a summary of all the preprocessing steps.

This pre-processing and the main pipeline evaluation can be applied to any data that have the same kind of properties as our data: biased (for our case it is biased by age), medium-sized (approximately 100 entries) datasets that need correction, extraction of meaningful features and protection from overfitting during feature selection.

## 3. Results

**Figure 12:**
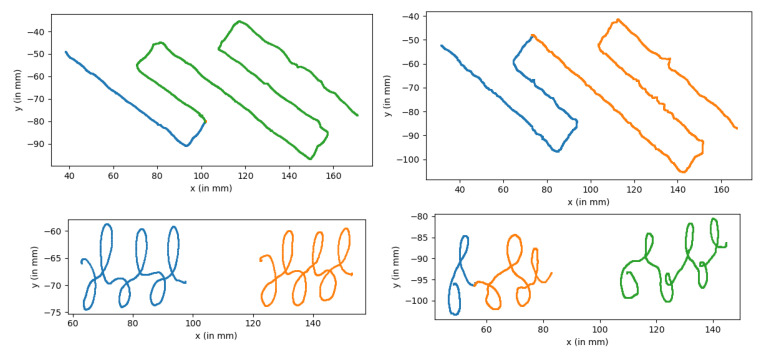
Stimuli performed by two children. Each color matches a different stroke. At the top: *Circuit 2*; at the bottom: *The loops*. The drawings on the left have been realized by G0210, 8 years old, in Grade 2 (TD) and the drawings on the right by D0024, 8 years old, in Grade 2 (DYS). We can see more tremors on the right than on the left.

The recording of the graphomotor tests allowed to highlight the differences between the drawings of children with and without dysgraphia. Examples are shown in Figure 12, where it can be noted that the number of strokes is higher in the DYS child compared to the TD child, and the trace is visually less regular. Features like “Number of angle changes” in *The Loops* and “Mean squared error to the path” in *Circuit 2* will have different values and help the correct classification of these children.

For the feature selection step, two of the six estimator candidates were selected for their compatibility with feature ranking: Linear SVM and Extra Trees. These estimators select different sets of features, leading to different final results for the subsequent following classification step. The features selected by each estimator are described in Table 4. Because this step is placed before the final classification, it was not dependent on the nature of the final estimator. That is why there are two different sets of features selected and not 18.

Because the train set changes for each fold of cross validation, and because the folds change for each iteration of cross validation (the random seed is different), the ten selected features may be different each time. Some features were selected every time, meaning they are highly discriminative and their selection does not depend on the presence of specific examples in the train set and, on the contrary, some features are rarely selected and most likely are discriminative only when certain examples are present in the train set. The percentage indicated next to the features in Table 4 represents the percentage of splits for which the feature has been selected. Because there is 100 cross-validation of each five splits, there is a total of 500 train/test splits for which the feature selection is conducted. The features shown in Table 4 are the ones selected in at least 200 splits.

The only features not selected by both estimators were the number of backtracking when drawing the third line of Circuit 1, the height without loops and the standard deviation of the velocity peaks when drawing The Loops (selected by the Linear SVM and not Extra Trees), and the length of the horizontal diameter of the circle in Shapes 1. Both did not select any features from Shapes 2 or Circuit 3, either because they were not discriminant enough or because they were too correlated to other features and discarded during the first pipeline step.

The other difference between both estimators used for Feature Selection is the consistency of the selected features between the splits. Eleven features were selected for more than 40% of the splits by the SVM, and even six more than 90% of the time, whereas for the Extra Trees only nine features were selected for more than 40% of the splits, and only one is almost always selected (Mean squared error during the short parts of the circuit (99.2%)). This indicates that Extra Trees, as a mean of feature selection, is less consistent throughout the different train/test splits of the cross validation, and more sensible to some data, and so possibly more prone to the selection of features that match noise rather than actual pattern in the drawings of children with dysgraphia. This reflects in the general classification results as well. The accuracy, sensitivity (also called recall) and specificity obtained by cross-validation are lower when feature selection is conducted by Extra Trees than by Linear SVM.

As most selected features come from *The Loops*, it seems to be a task of high interest for qualifying dysgraphia. With the exception of “Std of the velocity peaks” and “SNR on y axis”, all features selected are features specific to one test, and not general features that would be computed for all stimuli. This implies it is the diversity and specificity of the graphomotor tasks that reveal the characteristics of dysgraphia, rather than a general characteristic of the drawings.

The performances of the different pipelines configurations are listed in Table 5. As said above, when using Extra Trees for feature selection, the final classification has lesser results than when using a Linear SVM. Six models were eliminated, the three remaining have the same range of performance. They are: Random Forest (RF), Extra Trees (ET) and a Multi-Layers Perceptron (MLP). The accuracy was the main metric used for the selection, but the sensitivity and specificity were computed as well. The best accuracy score is 73.4% obtained with RF with 500 estimators. The best configuration of MLP (one hidden layer of size 12) gives 73% accuracy and it is the same result for ET with 500 estimators. The main difference between the three models is the repartition between Sensitivity (True Positive Rate) and Specificity (True Negative Rate). A higher sensitivity means that fewer children with dysgraphia remain undetected, and a higher specificity means that fewer typically developing children are misclassified by the algorithm.

## 4. Discussion

An accuracy of 73% has been reached in this study, which may seem to be relatively low compared to previous studies using text data, as seen in Table 1. However, it is a promising result for the use of non-text-based data for this kind of prediagnosis tool. The studies presented in Table 1 have access to text data, and our goal was to show that classification was possible without such data. Thus, with the new specific features extracted and a pipeline focused on reducing overfitting, we could afford not having any text-based data and still reach 73% accuracy on the classification of dysgraphia, a writing disorder.

The accuracy of other estimators, such as SVM or Ada boost, may also seem low when compared to other studies in unrelated fields such as [40]. It seems, for SVM, that the data is hard to separate, even when projected into other spaces (such as with the RBF Kernel).

We used cross-validation because we could not afford having a completely separated train set, due to the low amount of subjects with dysgraphia (43). Cross-validation remains a solid method for validating such a model and avoiding overfitting. Any pre-processing steps were either conducted using separated data (for the Z-score) or as a part of the pipeline (correlation filtering, feature selection) in order to prevent any overfitting due to test data leakage in the train set.

Very few features were selected (10 out of 345) for each evaluation, but some of them may still have been selected because of statistical noise in the data. This suspicion is particularly important when the features selected vary highly between the different splits of the cross-validation, because it means the features are selected only due to the presence of particular examples and most likely will not help the generalizability (applicability to new datasets) of the pipeline, as is the case when Extra Trees are used for the selection. This kind of suspicion can be controlled in a future analysis by evaluating a model built using only the features selected presented in Table 4 with new test data.

One of our goals with feature selection and the initial feature extraction was to have a very explicative model. That is why we did not use automatic feature extraction or PCA [41]. However, it must be noted that because the first step (suppression of correlated features) will select arbitrarily between two correlated features, it may deteriorate the explicative part of the model in some way. This is a minor issue because even if the two correlated features are different and one of them is randomly not selected after the first step, the fact that they are highly correlated (at least 85%) means that they do not essentially represent different particularities of the drawings, so this choice will not change the final interpretation of the characteristics of dysgraphic drawings. Overall, these characteristics are typical of dysgraphic handwriting, but transposed to the context of graphomotor tasks, especially for *The Loops*, which is close to a real writing task.

Features related to the detection of Parkinson’s Disease were used, along with *The Loops*, a test used to quantify the severity of parkinsonian dysgraphia [29,37,42]. It is crucial to note that parkinsonian and developmental dysgraphia probably do not result from the same neurological causes, even if they can share some similar effects on the handwriting (their main difference being parkinsonian micrographia [42]). Yet, including these features and this test seemed relevant to our study. Indeed, five out of eleven of the most selected features come from *The Loops*, meaning that its characteristics show differences between typically-developing children and children with dysgraphia that the other graphomotor tests cannot see, thus they are complementary. It is not that surprising, given that *The Loops* is the test closer to actual writing among the six graphomotor tests used here. The inclusion of a test close to handwriting was indeed important in our case (detection of developmental dysgraphia and not motor difficulties), since not all dysgraphia subtypes impair drawing [43].

We had to withdraw the features related to pen pressure due to calibration issues between the tablets. Adding them could improve the performance of the classifier [11,12]. It would be interesting to consider those in a future analysis by using the same graphic tablet model for all data acquisitions, or with a good method to calibrate different graphic tablets.

In terms of performances, with an accuracy of 73% for the best model, this study presents lower results than the previous one using graphomotor tests, which reported an 84% accuracy [21]. The main difference in our approach is not the size of the datasets (26 healthy children, 27 with graphomotor difficulties), but the nature of the subjects and the goal of the study: detection of dysgraphia using only drawing tests for this study, and detection of self-reported graphomotor difficulties for Galaz et al., using an adapted set of tests. Furthermore, their study was more focused on feature extraction and quality control (more features were extracted in their study), rather than on model selection and a focus on the explicative part of the final model. A difference in methodology might also explain the performance gap between the two models. They used Sequential Forward Feature Selection (SFFS) [28], which trains a second model to select the features. However, it is not clear if they preprocessed their features before the feature selection or as a part of the feature selection. As reported in their paper, it seems that they used the second option which, in this case, can lead to some overfitting, as SFFS will perform cross-validation on biased data.

The participants were all in the 2nd Grade at least, because we needed them to have at least two years of experience in handwriting in order to be rated by the BHK test and have labels for the model to learn with. In the view of conceiving a pre-diagnosis tool that would be targeting younger children that do not know how to write yet, a follow-up study could make children perform the graphomotor tests at an earlier age and then make them take the BHK test a few years later, when they would be able to. This way, it would be possible to estimate whether at-risk children detected by the model actually developed dysgraphia, and which features were the most useful for this detection.

Another goal of this study was to provide a more objective assessment of the childrens’ drawing skills and detect if they are at risk for developing dysgraphia. The subjectivity part in the examiner’s ratings has been evidenced by [6] when establishing their standard for the BHK test. They showed that there was a variance in the examiners’ ratings. The correlation between the BHK score of the examiners ranges from 0.68 to 0.90 (respectively inexperienced and experienced raters) which may lead to divergences in the given BHK score and consequently in the diagnosis for a same child. In this study either one or two experienced examiners, who then gave their diagnosis, rated each BHK test. It did not necessarily perfectly match the threshold at two standard deviations from the mean: This means that children with a BHK test score above −2 (above the threshold at which the diagnosis is supposed to be made) could be considered as dysgraphic, because other contextual information (such as the quality of the writing at school) was considered as well. It is worth considering that other examiners could rate differently and that some labels may then be inaccurate for some children. However, in our active database, this could only concern 8 children out of 305, who are close to the cutoff, with a BHK score comprised between −2.25 and −1.75.

Our machine learning based pre-diagnosis tool aims at avoiding this variability by providing an objective assessment of the childrens’ handwriting skills through not only the classification but also the drawing features involved in this classification. The fact that we can help in the detection of dysgraphia by specialists, without having any writing task and thus independent of the nationality of the subject, is the main feat presented here.

## 5. Conclusions

We proposed a pre-diagnosis tool for dysgraphia based on graphomotor tests only and machine learning models trained with data we collected and features we extracted. We designed a pipeline and pre-processing steps to use our data, reduce its bias, and select features without overfitting, in order to help the generalizability of our results. An accuracy of 73% was obtained on the classification test, which is a very encouraging result. The use of graphomotor tests to diagnose dysgraphia is quite new in the field and this study should pave the way to new possibilities for approaching dysgraphia without resorting to pure handwriting, making it independent from nationality. *The Loops*, a test particularly close to handwriting used also in Parkinson Disease qualification, was especially useful, providing half the features used in the end, indicating a good relevance for dysgraphia detection. Three models seem to present good results in classification when used in our pipeline: Random Forest, Extra Trees and Multi-Layer Perceptron. Our general database is exhaustive and thus allowed us to reduce the bias due to age regarding writing and its development. The extracted features cover a broad part of dysgraphia’s criterions and handwriting kinematic and are easy to interpret. Even if dysgraphia is not a binary characteristic and keeping in mind that a classification might be biased, such a pre-diagnosis tool could allow operating an early pre-selection of the at-risk children, with drawing tests only, leading to early intervention on handwriting difficulties and a general improvement of the well-being of these children.

## Figures and Tables

**Table 1:** A summary of the different classification algorithms used in the last studies using text data only as training data. Some studies tried several models of classification of children with dysgraphia (DYS) and typically developing children (TD). ^1^ the score used is not balanced accuracy but only the percentage of children with dysgraphia actually detected by the algorithm. ^2^ the score used is the accuracy, because the sets are almost balanced.

Reference	DYS/TD Repartition	Model Used	Score
[11]	27/27	Random Forest	96.43%
[12]	56/242	Random Forest	97.9%
[13]	48/43	SVM (RBF Kernel)	82.51%
[16]	24/971	RNN	90% ^1^
[13]	42/36	Random Forest	67%
[14]	42/36	SVM	66%
[14]	42/36	AdaBoost	64%
[15]	57/63	Random Forest	77.6% ^2^
[15]	57/63	SVM	78.8% ^2^
[15]	57/63	AdaBoost	79.5% ^2^
[20]	122/458	SVM (RBF Kernel)	83%

**Table 2:** Description of the database, organized by diagnosis. For each group (TYP and DYS), the general characteristics, the BHK quality score (evaluation of handwriting) and the BHK speed score (evaluation of speed) are listed, with the *p*-value of the differences between both groups. A chi-squared test was used for gender, and a Welch test for the other categories to obtain these *p*-values.

	Typical (*n* = 262)	Dysgraphic (*n* = 43)	*p*-Value
**Age** (in years): mean (*std*)	8.95 (*1.22*)	9.20 (*1.15*)	0.20
**Gender**: Female/Male	120/142	11/32	**<0.02**
**Laterality**: Left-Handed/Right-Handed	33/229	2/41	
**Origin**: School/Hospital	252/10	13/30	
**BHK quality score**: mean (*std*)	**12.2** (*4.5*)	**24.2** (*5.8*)	**<1.10^−13^**
Grade 2: mean (*std*)	*n* = 89; **13.9** (*5.3*)	*n* = 7; **29.6** (*7.2*)	**<0.02**
Grade 3: mean (*std*)	*n* = 66; **11.7** (*4.0*)	*n* = 13; **24.7** (*3.6*)	**<1.10^−7^**
Grade 4: mean (*std*)	*n* = 57; **11.9** (*3.3*)	*n* = 15; **23.4** (*6.1*)	**<1.10^−4^**
Grade 5: mean (*std*)	*n* = 50; **10.2** (*3.8*)	*n* = 8; **20.3** (*2.9*)	**<1.10^−3^**
**BHK speed score**: mean (*std*)	**206** (*79*)	**161** (*89*)	**<0.01**
Grade 2: mean (*std*)	*n* = 89; **134** (*37*)	*n* = 7; **123** (*41*)	0.62
Grade 3: mean (*std*)	*n* = 66; **190** (*42*)	*n* = 13; **131** (*92*)	0.07
Grade 4: mean (*std*)	*n* = 57; **245** (*52*)	*n* = 15; **148** (*40*)	**<1.10^−6^**
Grade 5: mean (*std*)	*n* = 50; **305** (*62*)	*n* = 8; **275** (*100*)	0.53

**Table 3:** The general features extracted from all stimuli. All duration features are in seconds, length features are in cm, and velocity features in cm/sec.

Feature	Reference
Duration of the Stimulus	[31]
Writing Time	[31]
Mean, Median and Maximum Duration of a Stroke	[32]
Mean, Median and Maximum Duration of a Lift	[32]
Number of Strokes	[33]
On Air/On Paper Time Ratio	[11]
Duration and Length of the Paths Performed at Low Velocity (less than 1 mm in 150 ms)	[11]
Mean on Paper Velocity	[11]
Average Normalized Jerk	[11]
Number and Duration of Abnormal Stops	[34]
Number of Peaks of Velocity	[35]
SNvpd	[36]
Rényi Entropy of order 2	[37]
Signal-to-Noise Ratio	[37]

**Table 4:** The features selected. Each feature is followed by the percentage of times the feature was selected, the higher it is, the more important and generalizable the feature. No features from *Circuit 3* and *Shapes 2* were selected more than 40% of the time.

	Estimator Used for the Feature Selection Step	
Stimuli	Linear SVM	Extra Trees
Circuit 1	Number of backtrackings in the third line (44.8%) Percentage of the third line completed after the first stop (41.4%)	Percentage of the third line completed after the first stop (50.8%)
Circuit 2	Mean squared error during the short parts of the circuit (100%) Std of the slopes for the short parts of the circuit (99.6%) Std of the velocity peaks (96.2%)	Mean squared error during the short parts of the circuit (99.2%) Std of the velocity peaks (77.6%) Std of the slopes for the short parts of the circuit (65.2%)
Shapes 1	Ratio between the horizontal and the vertical diameters of the circle (98.8%)	Ratio between the horizontal and the vertical diameters of the circle (58.8%) Length of horizontal diameter (42.2%)
The Loops	Std of the height of the loops (98%) SNR on the y axis (90.8%) Number of changes in angle (72%) Height without loops in the drawing (51.8%) Std of the velocity peaks (51.6%)	SNR on the y axis (77.8%) Height without loops in the drawing (72%) Std of the height of the loops (65.2%)
Circuit 3	None	None
Shapes 2	None	None

**Table 5:** This table gives the performances when using nine different estimators, and feature selection is conducted using either Extra Trees (left column) or Linear SVM (right column). Acc. stands for accuracy, Sen. for Sensitivity (or recall) and Spe. for Specificity. The three highlighted results are the three top models using accuracy as the main metric.

		*Model for Selection*			
		Extra Trees		Linear SVM	
*Model for Estimation*		*Mean*	*Std*	*Mean*	*Std*
	*Acc.*	0.690	0.037	**0.734**	0.034
**Random Forest**	*Sen.*	0.706	0.055	0.751	0.046
	*Spe.*	0.674	0.052	0.717	0.043
	*Acc.*	0.700	0.038	**0.730**	0.033
**MLP**	*Sen.*	0.708	0.054	0.739	0.040
	*Spe.*	0.693	0.052	0.721	0.048
	*Acc.*	0.696	0.034	**0.730**	0.031
**Extra Trees**	*Sen.*	0.735	0.047	0.761	0.039
	*Spe.*	0.657	0.052	0.698	0.048
	*Acc.*	0.669	0.037	0.708	0.030
SVM (linear)	*Sen.*	0.564	0.070	0.615	0.053
	*Spe.*	0.773	0.057	0.801	0.041
	*Acc.*	0.506	0.012	0.506	0.010
SVM (polynomial)	*Sen.*	0.432	0.059	0.423	0.049
	*Spe.*	0.581	0.053	0.589	0.043
	*Acc.*	0.530	0.021	0.541	0.020
SVM (sigmoid)	*Sen.*	0.495	0.048	0.512	0.046
	*Spe.*	0.566	0.032	0.569	0.033
	*Acc.*	0.531	0.020	0.541	0.020
SVM (rbf)	*Sen.*	0.496	0.047	0.513	0.046
	*Spe.*	0.566	0.032	0.569	0.033
	*Acc.*	0.671	0.039	0.703	0.035
Gaussian Naïve Bayes	*Sen.*	0.585	0.064	0.605	0.048
	*Spe.*	0.757	0.049	0.802	0.046
	*Acc.*	0.646	0.048	0.690	0.041
Ada Boost	*Sen.*	0.659	0.064	0.720	0.055
	*Spe.*	0.633	0.063	0.659	0.054

## Data Availability

The data presented in this study are available on request from the corresponding author. The data are not publicly available due to privacy and ethical reasons (medical data).
